# Angiogenesis-Related Gene Signature-Derived Risk Score for Glioblastoma: Prospects for Predicting Prognosis and Immune Heterogeneity in Glioblastoma

**DOI:** 10.3389/fcell.2022.778286

**Published:** 2022-03-18

**Authors:** Gang Wang, Jin-Qu Hu, Ji-Yuan Liu, Xiao-Mei Zhang

**Affiliations:** ^1^ Department of Neurosurgery, The First Affiliated Hospital of China Medical University, Shenyang, China; ^2^ Department of Rheumatology and Immunology, ShengJing Hospital of China Medical University, Shenyang, China

**Keywords:** glioblastoma, angiogenesis, gene signature, prognostic model, risk score

## Abstract

**Background:** Glioblastoma multiforme (GBM) is the most common malignant tumor in the central nervous system with poor prognosis and unsatisfactory therapeutic efficacy. Considering the high correlation between tumors and angiogenesis, we attempted to construct a more effective model with angiogenesis-related genes (ARGs) to better predict therapeutic response and prognosis.

**Methods:** The ARG datasets were downloaded from the NCBI-Gene and Molecular Signatures Database. The gene expression data and clinical information were obtained from TCGA and CGGA databases. The differentially expressed angiogenesis-related genes (DE-ARGs) were screened with the R package “DESeq2”. Univariate Cox proportional hazards regression analysis was used to screen for ARGs related to overall survival. The redundant ARGs were removed by least absolute shrinkage and selection operator (LASSO) regression analysis. Based on the gene signature of DE-ARGs, a risk score model was established, and its effectiveness was estimated through Kaplan–Meier analysis, ROC analysis, etc.

**Results:** A total of 626 DE-ARGs were explored between GBM and normal samples; 31 genes were identified as key DE-ARGs. Then, the risk score of ARG signature was established. Patients with high-risk score had poor survival outcomes. It was proved that the risk score could predict some medical treatments’ response, such as temozolomide chemotherapy, radiotherapy, and immunotherapy. Besides, the risk score could serve as a promising prognostic predictor. Three key prognostic genes (PLAUR, ITGA5, and FMOD) were selected and further discussed.

**Conclusion:** The angiogenesis-related gene signature-derived risk score is a promising predictor of prognosis and treatment response in GBM and will help in making appropriate therapeutic strategies.

## Background

Glioblastoma, also known as glioblastoma multiforme (GBM), which is classified by the World Health Organization (WHO) as a grade IV glioma, is a highly heterogeneous and aggressive type of nervous system tumor, with a 5-year survival rate of less than 7% (Ostrom et al., 2019). There has been great progress in surgical resection, radiotherapy, and chemotherapy. Immunotherapy, as a new promising treatment, has particularly caught worldwide attention (Bush et al., 2017; Xu et al., 2020). Nevertheless, the prognosis for GBM patients remains dismal.

Angiogenesis, which refers to the process of neovascularization from existing vessels, has been substantiated to be highly related to tumorigenesis, metastasis, and migration in glioblastoma (Onishi et al., 2011). Some angiogenesis regulators, including vascular endothelial growth factor (VEGF), basic fibroblast growth factor (bFGF), hepatocyte growth factor (HGF), platelet-derived growth factor (PDGF), transforming growth factor-β (TGF-β), matrix metalloproteinases (MMPs), and angiopoietins (Angs), are demonstrated to modulate several important cancer-related pathways and are promising prognostic biomarkers of GBM patients (Ahir et al., 2020). Inhibition of growth factors/signaling pathways necessary for tumor angiogenesis is viewed as one of the most practical approaches to hinder tumor progression (Ahir et al., 2020; Bazan et al., 2021).

Recently, with the advancement of next-generation sequencing technology, numerous studies have focused on the molecular changes underlying GBM. In 2016, for the first time, the World Health Organization (WHO) incorporated molecular marker-based classification into diagnosis, indicating that the treatment and diagnosis of GBM have entered a molecular era (Komori, 2017). Although increasing molecular studies in GBM have been reported recently, the appropriate prognostic biomarkers and predictors of therapeutic responses are still not clear. Increasing studies have investigated the roles of angiogenesis-related genes (ARGs) in the development and progression of glioma. The expression of ARGs is dysregulated in GBM and correlated with prognosis (Fei et al., 2015; Rostami et al., 2019; Simon et al., 2020). Therefore, ARGs are promising therapeutic targets and prognostic predictors in GBM.

In the present study, based on the global gene expression profile, we aimed to develop an angiogenesis-related gene expression signature and a nomogram model to predict prognosis and therapeutic targets in GBM. The related immunological features are also evaluated.

## Materials and Methods

### Gene Expression and Clinical Data Acquisition

**FIGURE 1:**
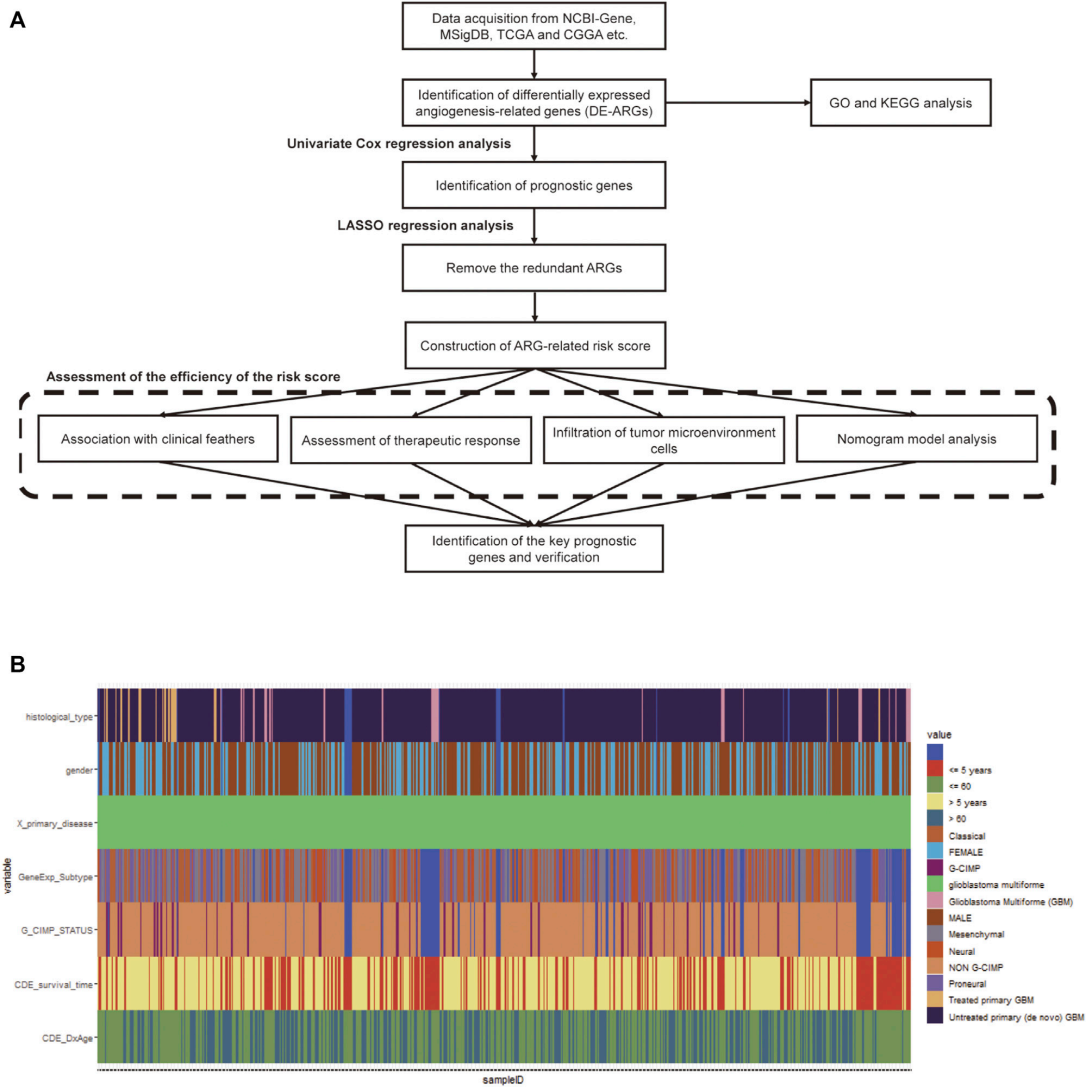
Workflow chart and the clinicopathological features of the data. **(A)** The workflow chart of the whole analysis in this study. **(B)** Heatmap of clinicopathological features of the TCGA-GBM data.

The study was carried out according to the workflow shown in Figure 1A. The ARG sets were downloaded from the NCBI-Gene (https://www.ncbi.nlm.nih.gov/gene) and Molecular Signatures Database (MSigDB, http://www.broad.mit.edu/gsea/msigdb). A total of 1,603 ARGs were obtained from NCBI-Gene with the keyword “angiogenesis” in *Homo sapiens* and 48 ARGs were downloaded from MSigDB (Supplementary Table S1).

The level III gene expression profiles and corresponding clinical information of GBM patients were downloaded from The Cancer Genome Atlas (TCGA, https://portal.gdc.cancer.gov) and the Chinese Glioma Genome Atlas (CGGA, http://www.cgga.org.cn) databases, respectively. The TCGA-GBM cohort containing 167 tumor samples and 5 normal samples was used as the training set (Figure 1B), while the CGGA cohort containing 388 GBM samples was selected as the validation set. A total of 219 GBM samples in The Repository for Molecular Brain Neoplasia Data (REMBRANDT, http://caintegrator-info.nci.nih.gov/REMBRANDT) and 159 GBM samples in GSE16011 database (https://www.ncbi.nlm.nih.gov/geo/) were also obtained and used as the validation set. The expression data regarding the efficacy of angiogenesis inhibitors in GBM was obtained from the GSE79671 database (https://www.ncbi.nlm.nih.gov/geo/). Protein–protein interaction (PPI) network data were obtained using the STRING database (http://string-db.org) (Szklarczyk et al., 2021). No ethical approval or informed consent was required in this study due to the public availability of the data.

### Identification of Differentially Expressed Genes and Functional Enrichment Analysis

The differentially expressed angiogenesis-related genes (DE-ARGs) between GBM and normal samples in the TCGA cohort were screened with the R language package “DESeq2”, using a cutoff of log2 fold change (log2FC) ≥1 and adjusted *p* ≤ 0.01 (Love et al., 2014). GO and KEGG pathway enrichment analyses were performed using the R package “clusterProfiler” (Yu et al., 2012).

### Development and Validation of Prognostic Signatures Based on ARGs

Univariate Cox proportional hazards regression analysis was used to screen for ARGs related to overall survival (OS). Then, the redundant ARGs were removed by least absolute shrinkage and selection operator (LASSO) regression analysis using the R package “glmnet”; thus, only 31 key ARGs remained (Friedman et al., 2009). The LASSO regression coefficients were weighted with mRNA expression levels to calculate the risk score.

By the risk score, patients were divided into high- or low-risk groups, respectively. With the R package “survival” and “survminer”, Kaplan–Meier survival analysis was carried out to compare the prognostic difference between the two groups, and then the results were verified by ROC analysis.

### Assessment of the Immune Landscape of GBM

In the present study, we analyzed the specific gene expression signature of immune and stromal cells in GBM tissues using the R package “estimate” (Yoshihara et al., 2013). By calculating the purity score, and immune and stromal scores with ESTIMATE algorithm, the infiltration of tumor microenvironment cells was predicted. Then, based on the normalized gene expression data, the proportions of 16 types of infiltrating immune cells were calculated by the CIBERSORT algorithm (Newman et al., 2015).

### Evaluation of the Prediction Efficiency of the Risk Score on Chemotherapy and Immunotherapy

We evaluated the distribution of the non-responding group and the responding group in high- and low-risk groups separately by analyzing the data from GSE79671. Tumor immune dysfunction and exclusion (TIDE) algorithm was used to predict tumor immune evasion (Jiang et al., 2018). The prognostic value on immunotherapy was verified by ROC analysis.

### Construction and Evaluation of the Nomogram

Furthermore, we plotted a nomogram based on the risk score groups and clinical traits by the multivariable Cox regression analysis. Then, validations were conducted utilizing the R package “rms” (version 6.2–0; http://cran.r-project.org/web/packages/rms). The calculation of concordance index (C-index) is to estimate the probability that the predicted result is consistent with the actual outcome.

### Identification of Key Genes

The network of 74 DE-ARGs was constructed by *Cytoscape* software (version 3.8.2) and the top hub genes were selected through the MCODE plugin. The prognostic value was examined by Kaplan–Meier survival analysis through the GlioVis data portal (http://gliovis.bioinfo.cnio.es) (Bowman et al., 2017). A Venn diagram analysis was carried out between the 31 key ARGs, 10 hub genes, and 8 prognostic hub genes previously identified; ultimately, 3 key genes were identified using venn tools in Hiplot (https://hiplot.com.cn) (Hiplot: A Free and Comprehensive Cloud Platform for Scientific Computation and Visualization, 2021).

### Statistical Analysis

Differences between the high- or low-risk groups were compared with the Wilcoxon test. Survival curves were generated by the Kaplan–Meier method and compared with the Log-rank test. Experiments were conducted three times independently and data were presented as mean ± SEM. *p* < 0.05 (*), *p* < 0.01 (**), and *p* < 0.001 (***) represent statistical significance. The time-dependent receiver operating characteristic (ROC) curves were built using the R package “pROC” to test the prognostic performance of the ARG-risk signature (Robin et al., 2011). All statistical analyses were conducted with SPSS 19.0 (IBM, Armonk, New York), GraphPad Prism 8.0 (GraphPad Software, La Jolla, California), or R software (www.r-project.org).

## Results

### Identification of Differentially Expressed Genes and Functional Enrichment Analysis

**FIGURE 2:**
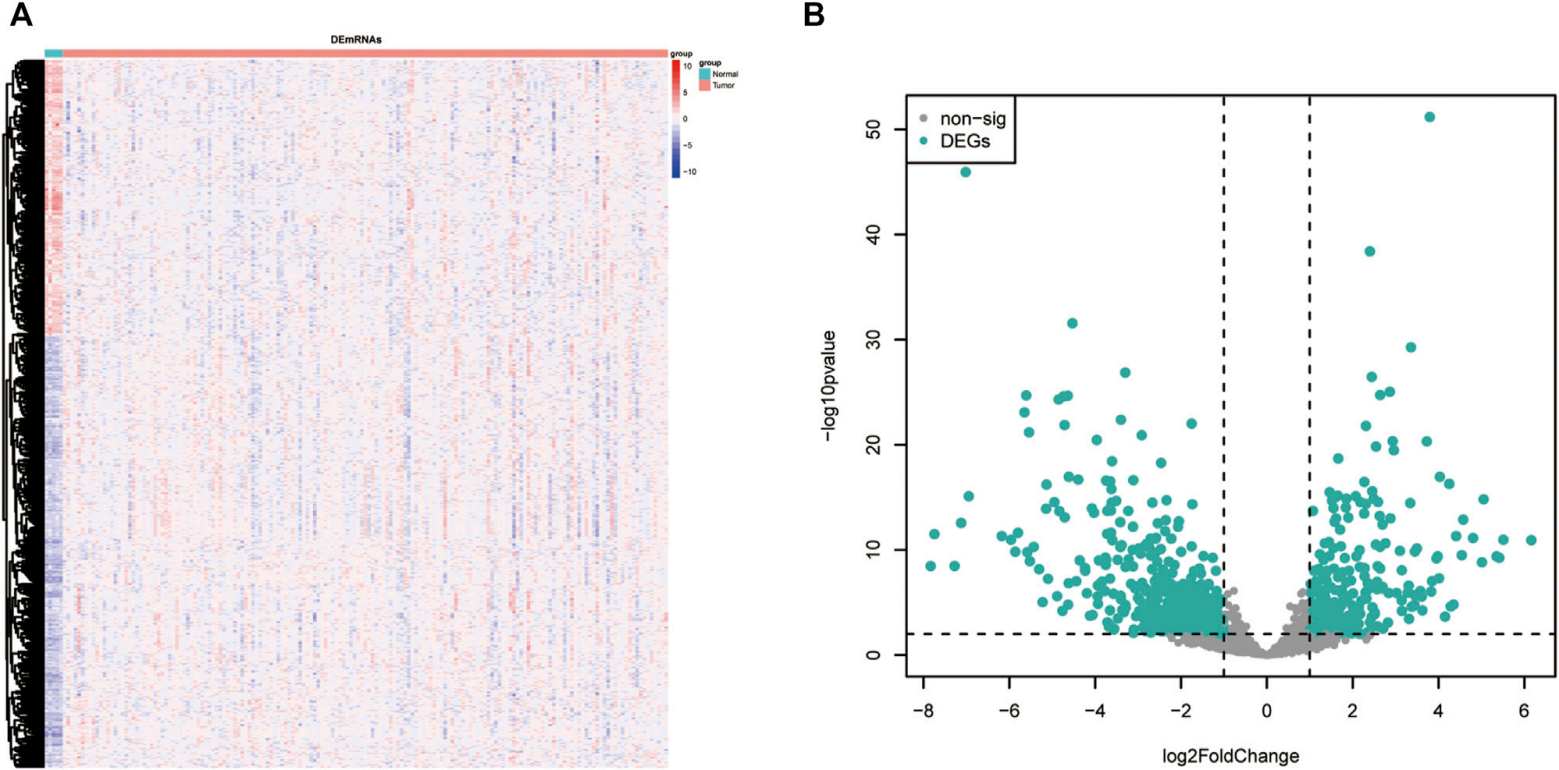
DE-ARGs between GBM and normal brain tissues. **(A)** Heatmap of the DE-ARGs. **(B)** Volcano plots presenting the differences between GBM and normal brain tissues. The blue dots represent DE-ARGs (adj. *p*-value < 0.01 and |log2(FC)| > 1). DE-ARGs, differentially expressed angiogenesis-related genes.

The TCGA-GBM dataset was screened to identify the differentially expressed angiogenesis-related genes (DE-ARGs) between GBM and normal samples with the R language package “DESeq2”, using a cutoff of |log2FC| ≥1 and adj.*p* < 0.01. As shown in Figure 2, there were 626 DE-ARGs in total; 243 genes were upregulated and 383 genes were downregulated (Figure 2).

**FIGURE 3:**
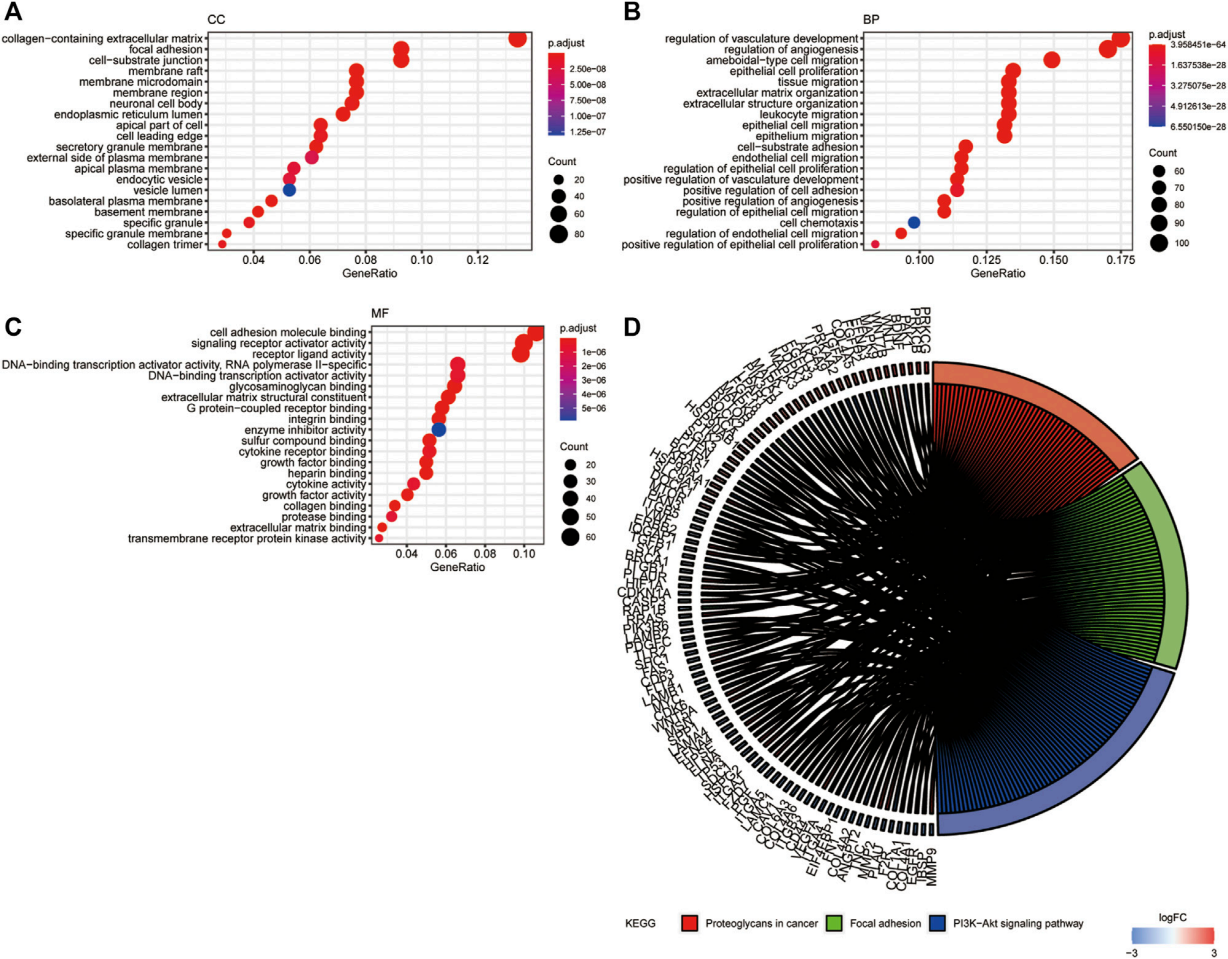
GO and KEGG functional enrichment analysis of DE-ARGs. **(A–C)** The top 20 enriched terms in CC, BP, and MF are presented. **(D)** The top 100 genes and 3 pathways enriched in KEGG analysis. GO, Gene Ontology; CC, cellular component; MF, molecular function; BP, biological process; KEGG, Kyoto Encyclopedia of Genes and Genomes. DE-ARGs, differentially expressed angiogenesis-related genes.

GO and KEGG pathway enrichment analyses were performed with a cutoff of *p* < 0.05. The top 20 most enriched terms are shown in Figure 3. GO analysis showed that the DE-ARGs were mainly enriched in cellular components like collagen-containing extracellular matrix, focal adhesion, and cell–substrate junction; biological processes like regulation of angiogenesis, regulation of vasculature development, and ameboidal-type cell migration; and molecular functions like cell adhesion molecule binding, signaling receptor activator activity, and receptor–ligand activity (Figures 3A–C). Meanwhile, KEGG analysis showed that the proteoglycans in cancer, focal adhesion, and PI3K-AKT signaling pathway were highly enriched. All the results suggested that the genes had a broad impact on tumor progression *via* angiogenesis regulation.

### Identification of Prognostic ARGs and Establishment of the ARG-Related Prognostic Model

**FIGURE 4:**
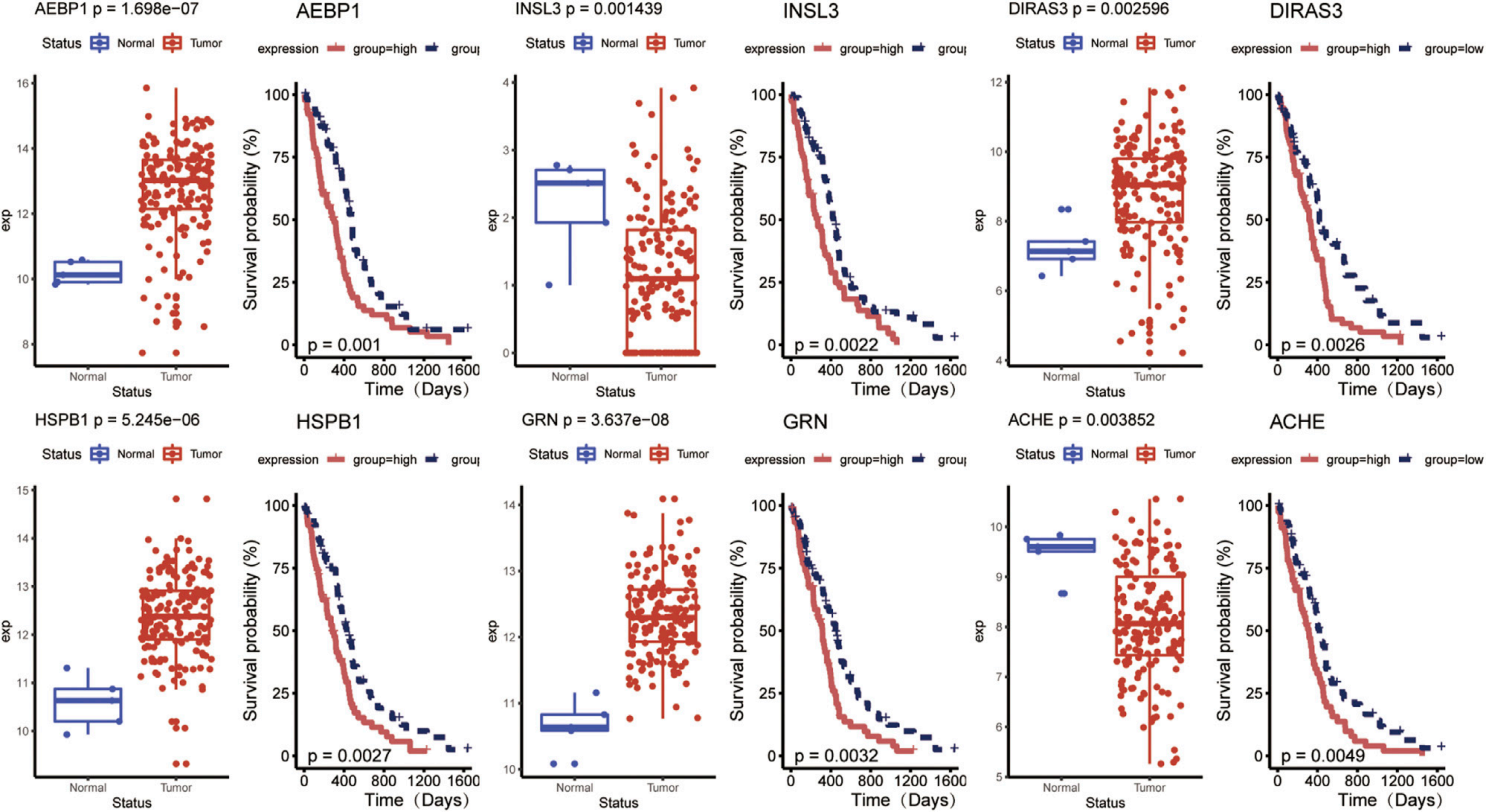
Top 6 prognostic DE-ARGs’ expression and their Kaplan–Meier survival curves. DE-ARGs, differentially expressed angiogenesis-related genes.

**FIGURE 5:**
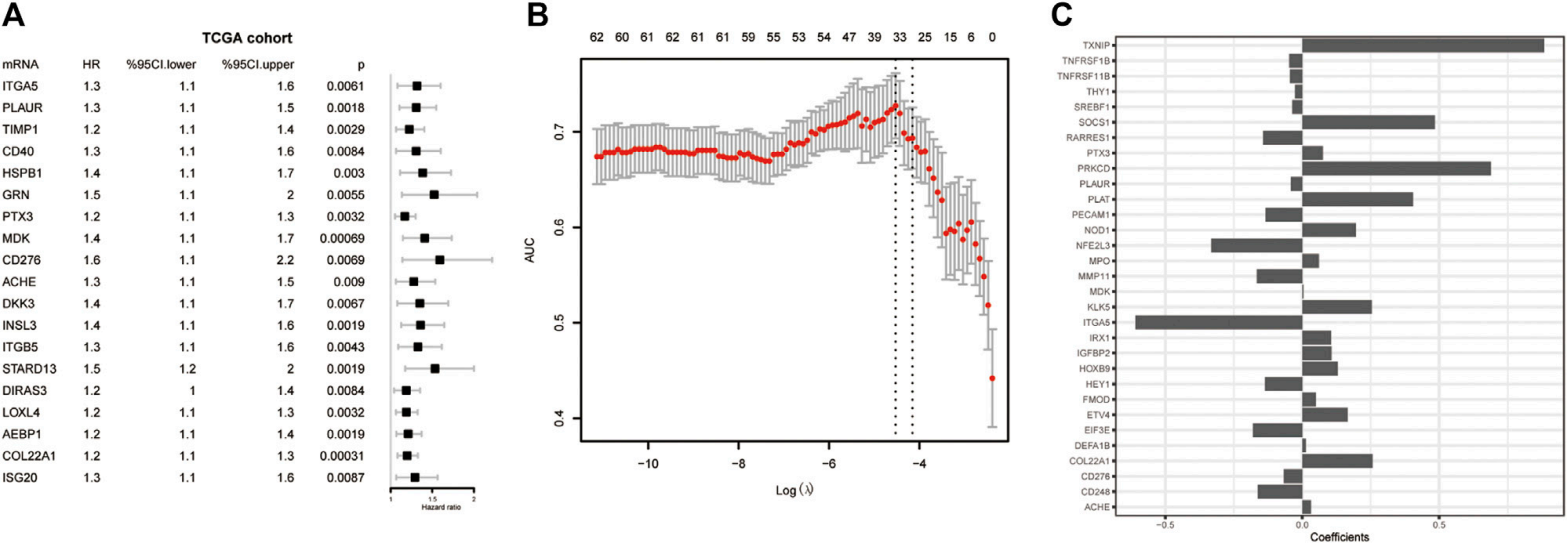
Screening for the key genes from the DE-ARGs. **(A)** Cox regression analysis. **(B)** LASSO penalized Cox regression analysis of the DE-ARGs. **(C)** The weight coefficient of key DE-ARGs. DE-ARGs, differentially expressed angiogenesis-related genes. LASSO, the least absolute shrinkage and selection operator.

Univariate Cox regression analysis was conducted to detect prognostic DE-ARGs. Seventy-four DE-ARGs were shown to be highly related with prognosis. The expression levels and OS curves of the top 6 are shown in Figure 4. Subsequently, 31 genes were selected as key factors from the above 74 DE-ARGs by LASSO Cox regression analysis (Figure 5).

With the 31 key factors, we established an ARG-related risk score model to predict the prognosis. The formula of the risk score of ARG signature reads as follows:

$$\mathrm{riskscore}\;=e^{\sum_{k=1}^{n}c_{k}\exp ression_{k}}$$

In the above equation, *n* is the number of selected key ARGs, *e* refers to the natural constant, *k* represents the *k*th prognostic gene with a non-zero dimension reduction coefficient of Lasso, *c* is its coefficient, and *expression*_*k*_ is its expression value.

**FIGURE 6:**
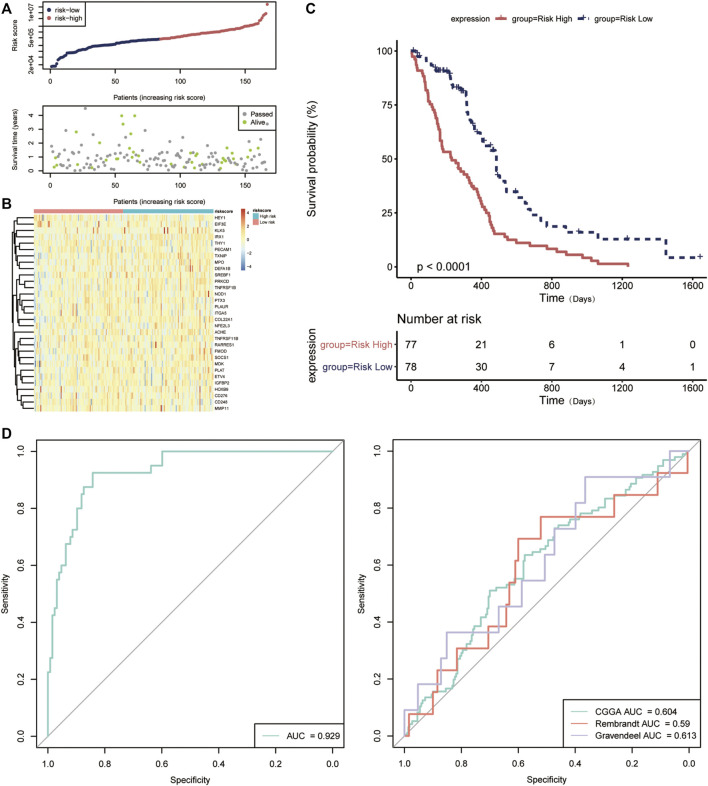
Validation of the prognostic value of the angiogenesis-related risk score model. **(A)** The distribution of the risk score model and patient survival status. **(B)** Heatmap presenting the expression of 31 key DE-ARGs in the high- and low-risk group classified by the risk score model. **(C)** Kaplan–Meier curves for overall survival in the high- and low-risk group. **(D)** The receptor operating characteristic (ROC) curve of the risk score in TCGA database (left) and other databases (right). AUC, area under the curve.

Hereafter, we sought to explore the relationship between the risk score and prognosis. We used the median value of the risk score as a cutoff to divide the patients into high- and low-risk groups (Figures 6A,B). Compared to the low-risk group, the high-risk group has a significant low OS, suggesting a worse prognosis (Figure 6C). Receiver operating characteristic (ROC) curve analysis showed that the ARG-related risk score had good predictive accuracy for prognosis in the TCGA cohort (Figure 6D). For independent validation, we further assessed the risk score model using the CGGA, Rembrandt, and Gravendeel database (Figure 6E). Consistent results were obtained.

### Association Between the Risk Score and Clinical Features

**FIGURE 7:**
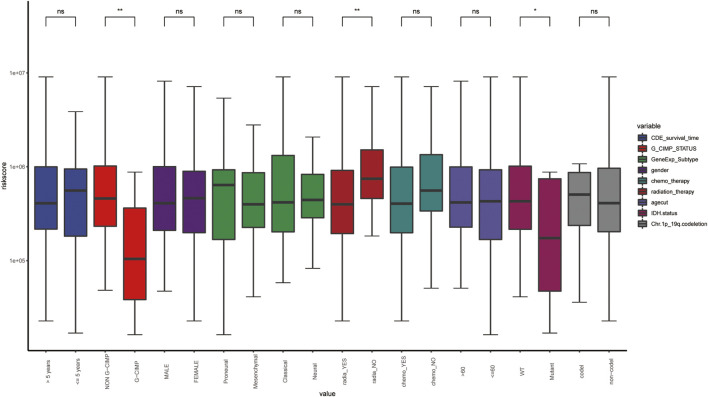
The distribution of clinical features in the different risk groups. WT, wild type; *, *p*-value < 0.05; **, *p*-value < 0.01; ns, non-significant.

In order to explore the correlation between ARG-related risk score and clinical features, we respectively compared the differences between the high- and low-risk groups in survival time, Glioma CpG island methylator phenotype (G-CIMP) status, gene expression subtype, isocitrate dehydrogenase (IDH) mutant status, etc. It was shown that the risk score did reveal a relationship with some clinical traits, especially in G_CIMP status and IDH mutant status (Figure 7).

### The Risk Score Predicts the Infiltration of Tumor Microenvironment Cells

**FIGURE 8:**
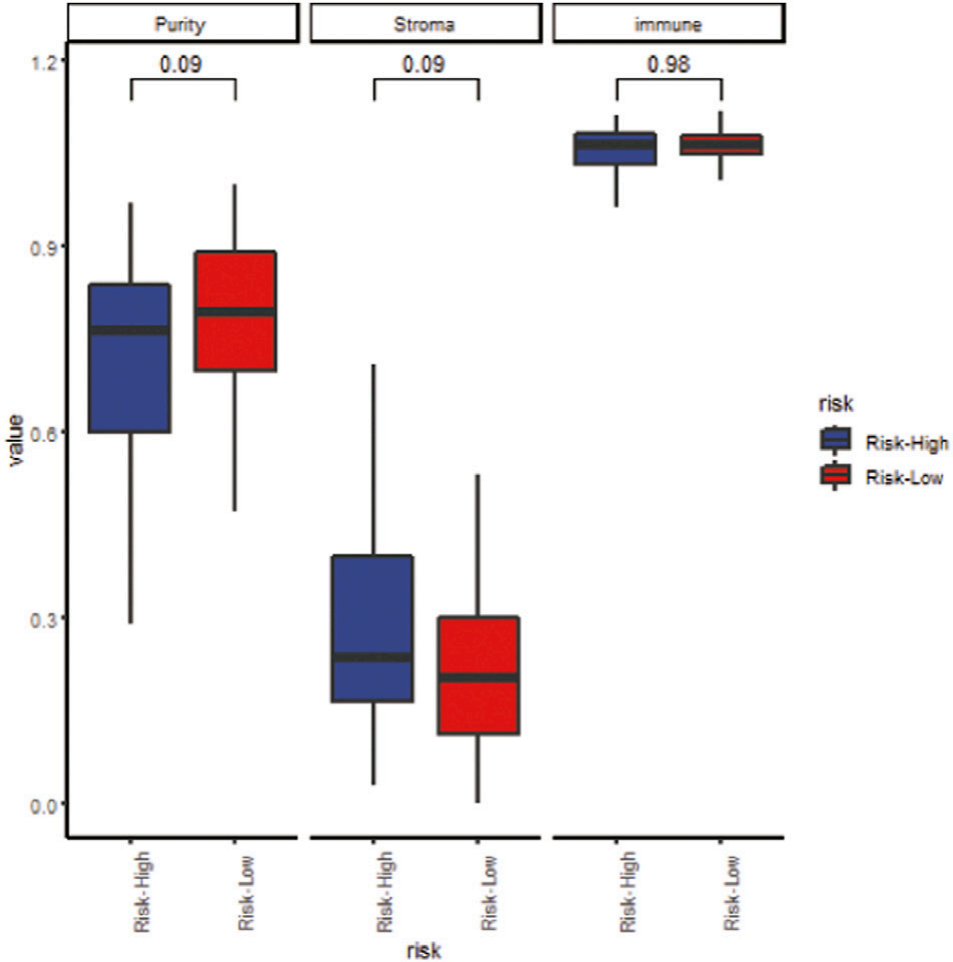
The correlation between the risk score and tumor purity (left), stroma score (middle) and immune score (right).

**FIGURE 9:**
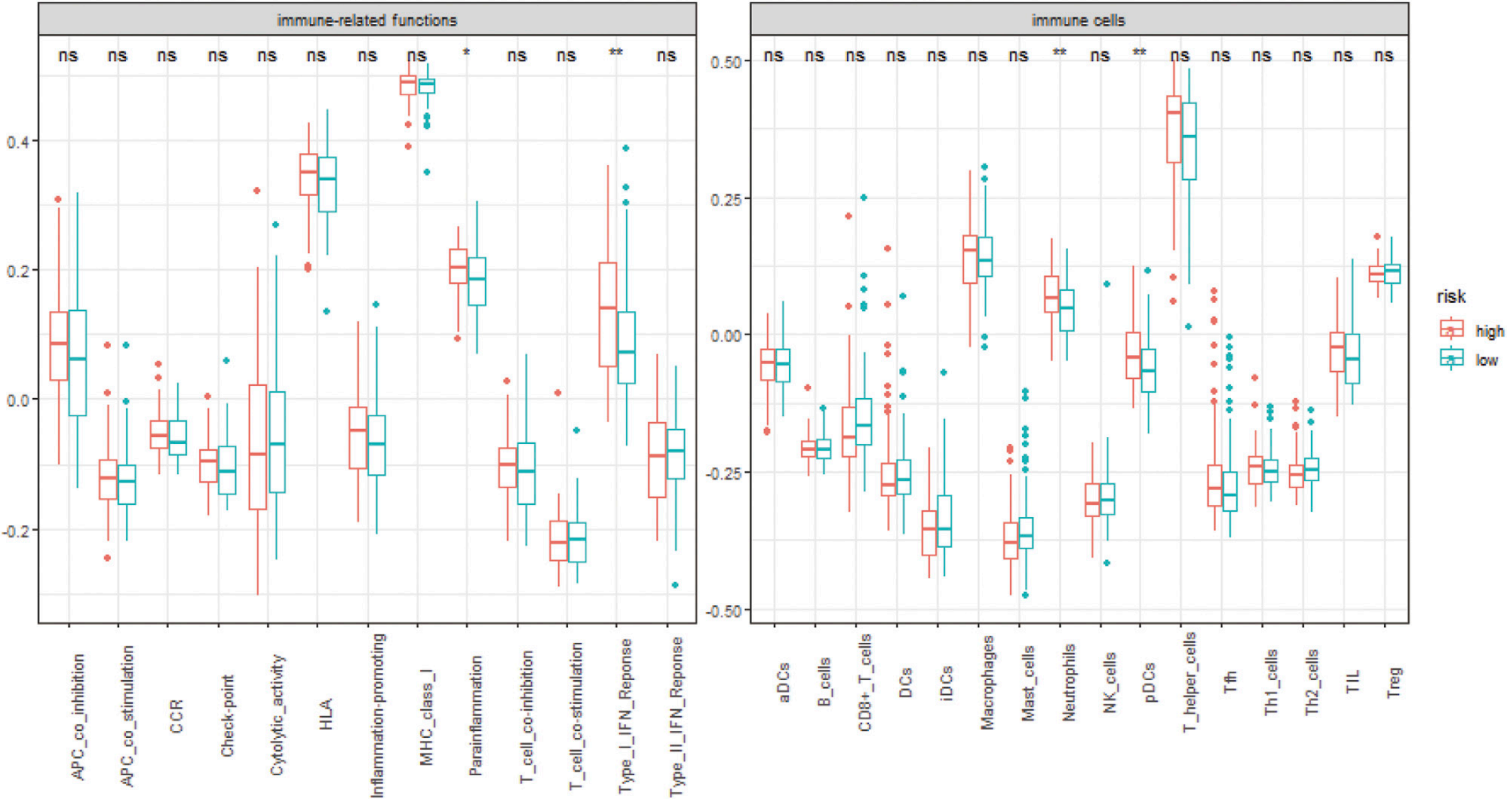
The distribution of 13 different immune-related functions and 16 types of infiltrating immune cell in the different risk groups. *, *p*-value < 0.05; **, *p*-value < 0.01; ns, non-significant.

We analyzed the specific gene expression signature of purity, immune, and stroma scores in GBM tissues using the R package “estimate”. As shown in Figure 8, there was no difference between the two groups in tumor purity. Even though the differences were not significant, the high-risk group did show a higher stroma score and immune score, indicating a poorer prognosis (Figure 8). To estimate the immunological functioning in the high- and low-risk groups, the proportions of 16 types of infiltrating immune cell and 13 types of immune-related functions were calculated by the CIBERSORT algorithm using the TCGA database. High-risk score was shown to be correlated with many immune-related functions, especially type I interferon (IFN) antiviral response and para-inflammation function. Furthermore, the distribution of immune cell also differed; the high-risk group had higher proportion of plasmacytoid dendritic cells (pDCs) and neutrophils (Figure 9).

### The Risk Score Predicts the Medical Treatment Response

**FIGURE 10:**
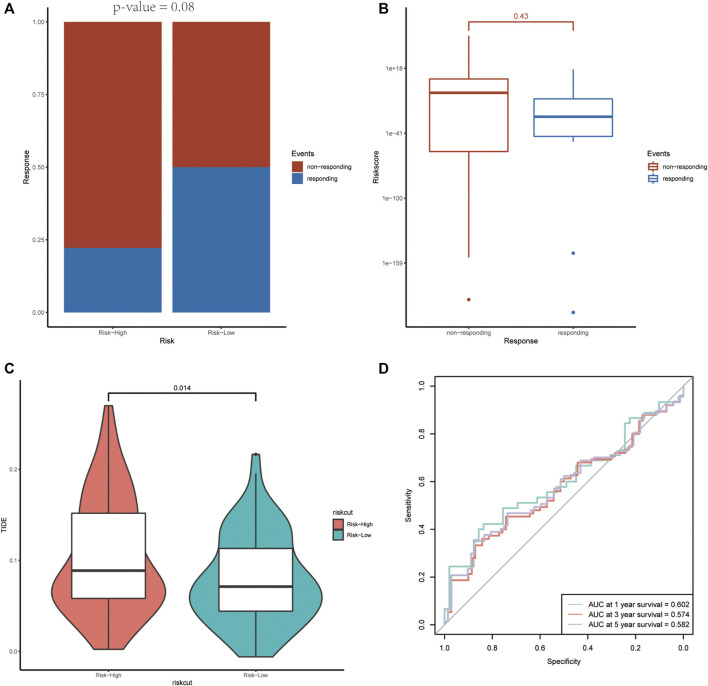
The association between the risk score and the treatment efficiency of anti-angiogenesis therapy and immunotherapy. **(A)** The responding ratio differs in the high- and low-risk groups. **(B)** The average risk score of the non-responding group was higher than that of the responding group. **(C, D)** The high-risk group revealed a higher TIDE score and the ROC curve demonstrated the prognostic value of the risk score on immunotherapy.

The targeted drug bevacizumab, which is well known as an anti-angiogenesis drug, has been proven effective on many malignant tumors. Nevertheless, not all the patients could benefit from it. In order to examine the prognostic ability on anti-angiogenesis treatment, the efficacy of bevacizumab was compared between the two groups. As shown in Figure 10, there were more patients who responded to the drugs in the low-risk score group. Meanwhile, the non-responding group has higher risk scores relatively (Figures 10A,B).

Predictive potential of immunotherapy responsiveness among the two groups in TCGA-GBM was estimated by TIDE algorithm. The high-risk group revealed a higher TIDE score (Figures 10C,D).

### The Risk Score Was an Independent Prognostic Predictor

**FIGURE 11:**
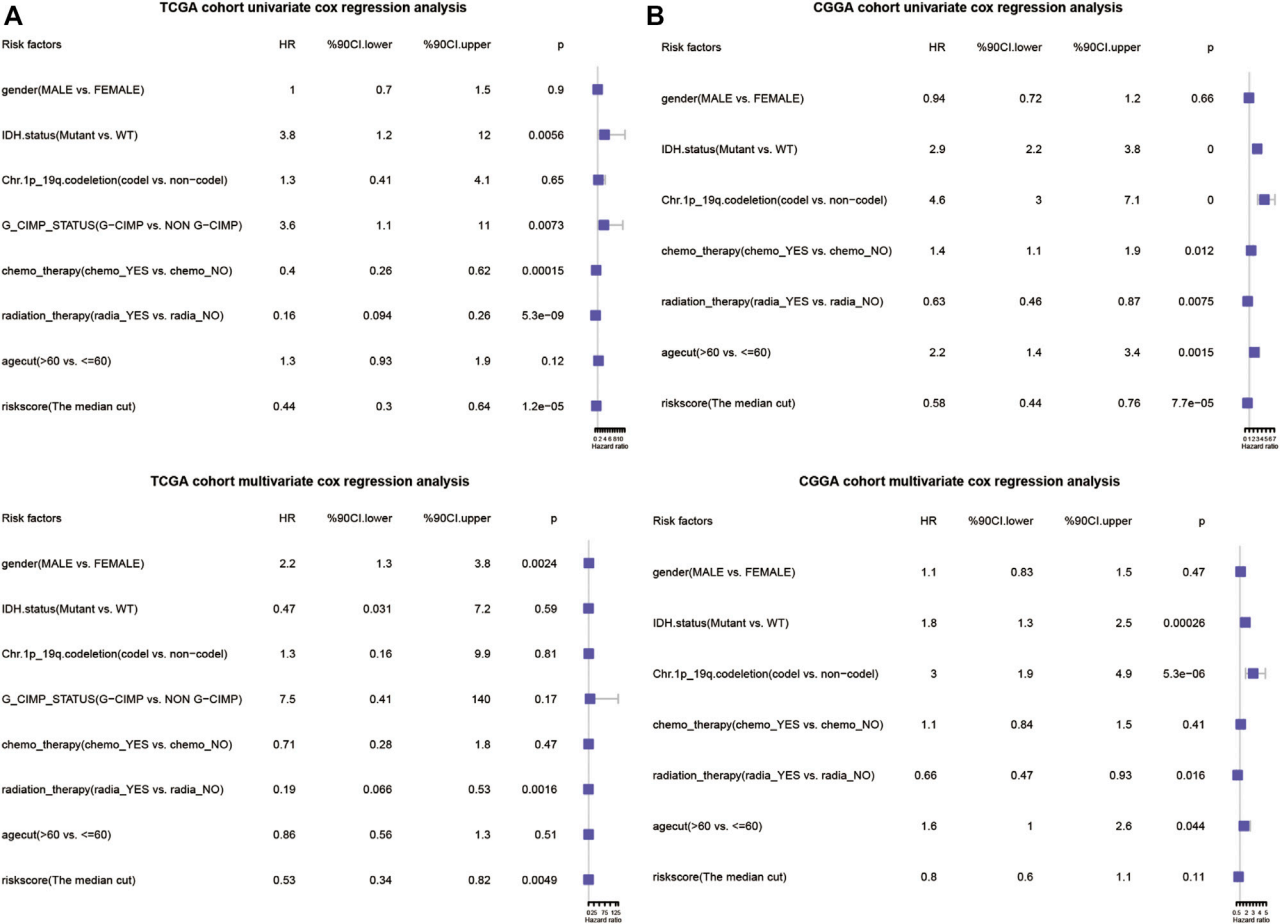
Forrest plots of the univariate and multivariate Cox regression analysis in TCGA database **(A)** and CGGA database **(B)**.

To access whether the angiogenesis-related risk score is a promising prognostic predictor, univariate and multivariate Cox regression was conducted with the TCGA-GBM dataset. The results revealed that the risk score, G-CIMP status, IDH mutant status, 1p/19q co-deletion, chemotherapy, and radiation therapy were significantly correlated with clinical outcome and prognosis (Figure 11). Similar results were obtained with the CGGA dataset. Taken together, the angiogenesis-related risk score was validated to be an independent prognostic predictor.

### Build Nomogram Combined the Risk Score With Clinical Features

**FIGURE 12:**
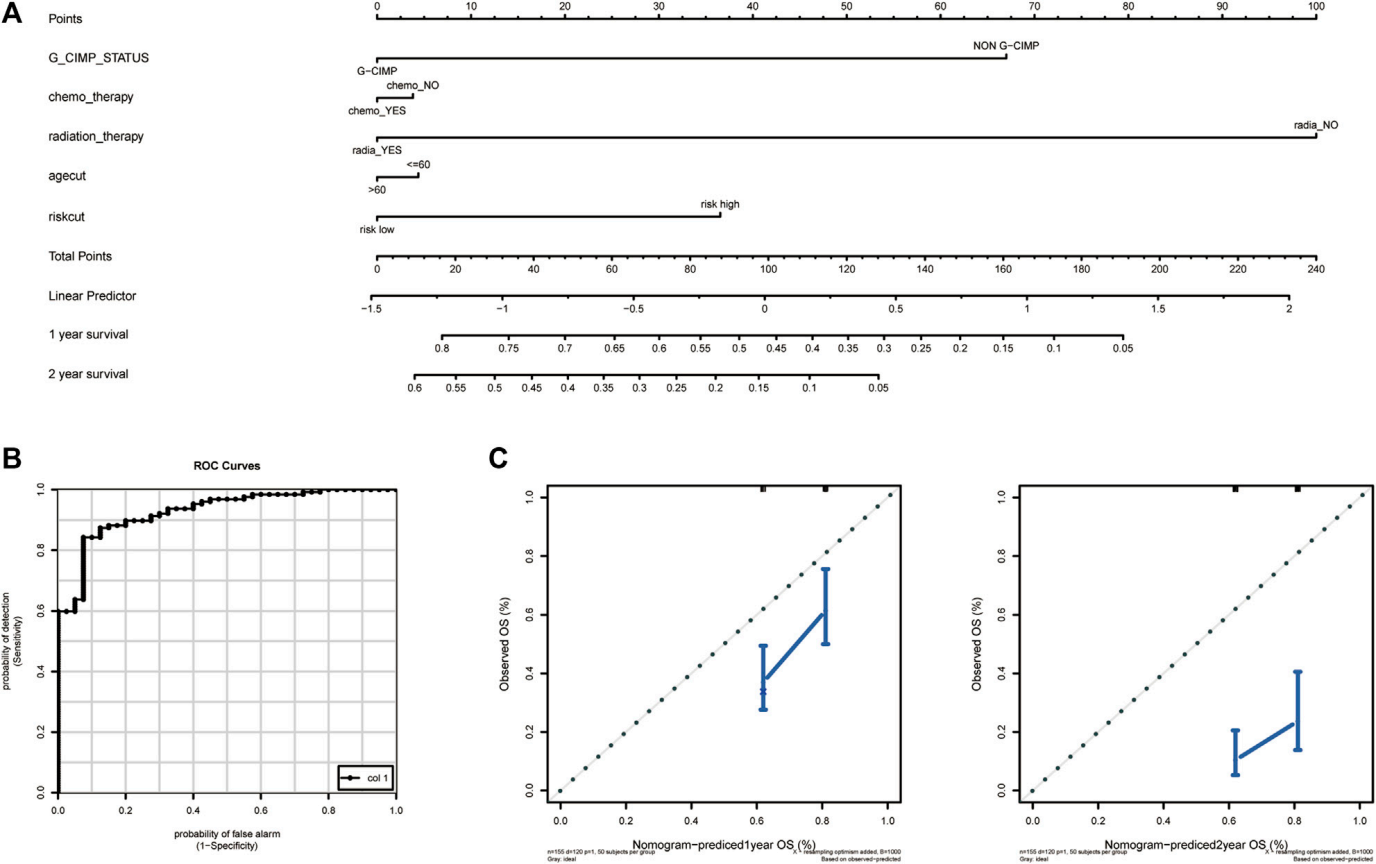
Establishment and validation of the nomogram to predict the prognosis. **(A)** Calibration plot of nomogram. **(B)** The ROC curve of the risk score. **(C)** The efficiency of nomogram on predicting 1 year OS (left) and 2 year OS (right). OS, overall survival.

In order to explore a new model for predicting, a nomogram was generated with the above independent prognostic clinical features to predict the probability of the 12‐ and 24‐month OS in the TCGA cohort (Figure 12A). As is shown in Figure 12, the predicted OS was closely related to the actual OS (Figure 12B).

### Identification of the Key Prognostic Genes

**FIGURE 13:**
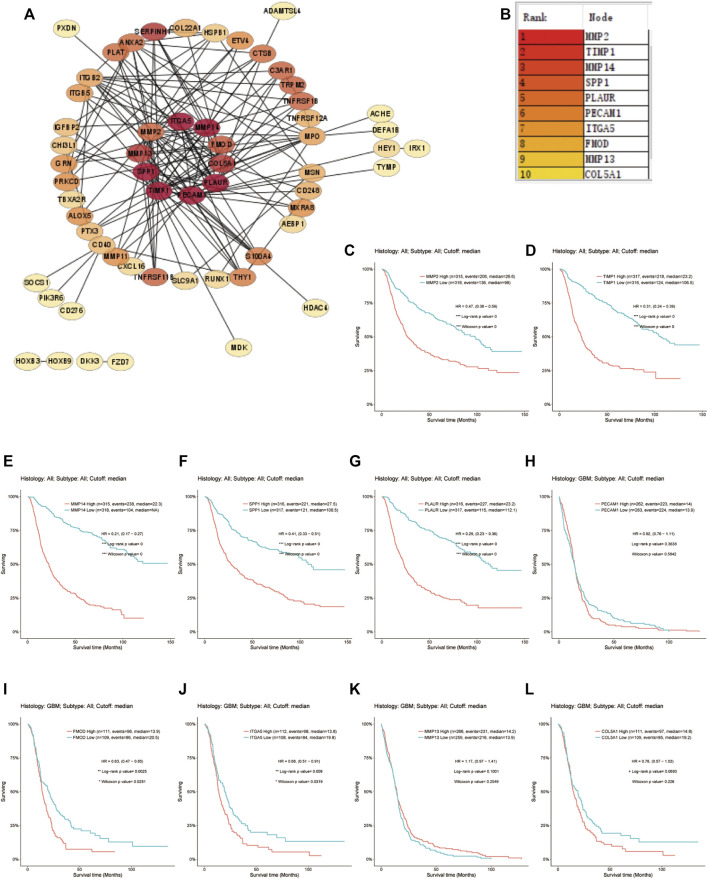
Screening for the hub genes. **(A)** PPI network of the DE-ARGs. **(B)** The top 10 hub genes selected by MCODE algorithm. **(C–L)** The correlation between the expression of hub gene and OS. PPI, protein–protein interaction; DE-ARGs, differentially expressed angiogenesis-related genes; OS, overall survival.

**FIGURE 14:**
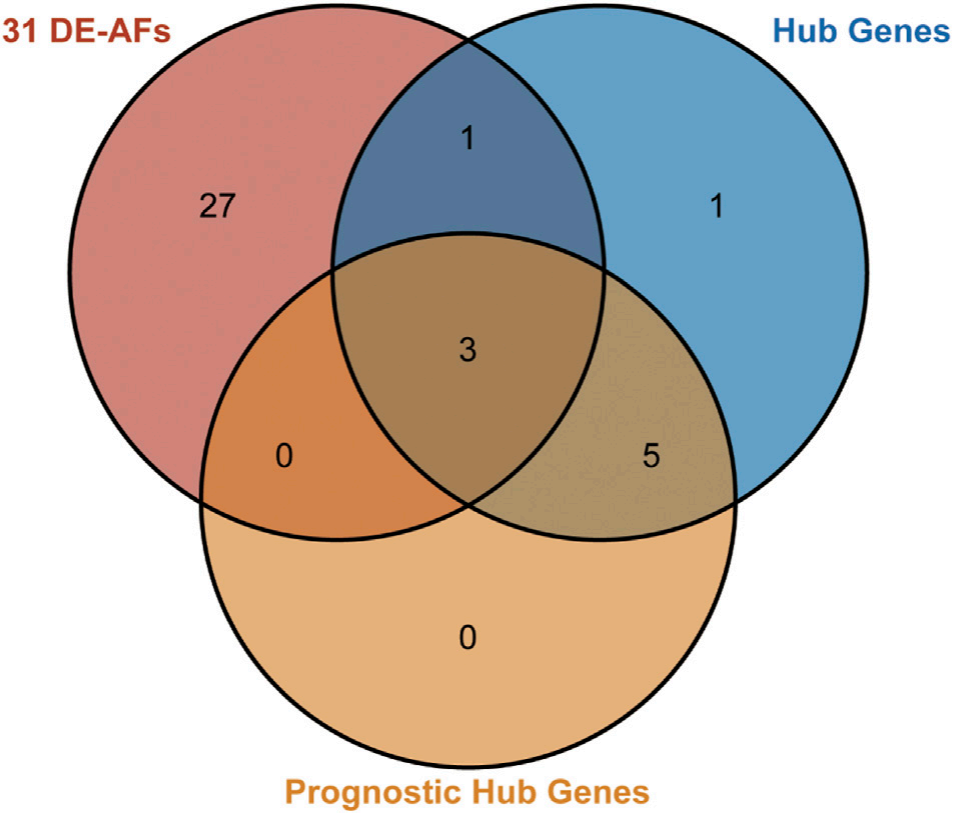
Venn diagram for identifying the key factors. The pink circle represents the 31 DE-ARGs. The blue circle represents the 10 hub genes. The orange circle represents the 8 prognostic genes selected by Kaplan–Meier survival curves. The overlap area represents the 3 genes that were predicted to play central roles.

The prognostic 31 DE-ARGs’ interaction network was constructed by *Cytoscape* software (Figure 13A), and the top 10 hub genes were identified through the MCODE plugin (Figure 13B). The Kaplan–Meier survival analysis was conducted and 8 genes were shown to be prognostic on OS (Figures 13C–L). Venn diagram analysis indicated that 3 key genes [PLAUR (plasminogen activator, urokinase receptor), ITGA5, and FMOD (fibromodulin)] were the intersection of the 31 DE-ARGs, 10 hub genes, and 8 prognostic hub genes (Figure 14).

## Discussion

GBM is a highly heterogeneous malignant tumor, and despite the great advances in multimodality therapy, the overall prognosis remains poor. Recently, numerous studies have focused on the molecular changes underlying GBM, supplying abundant high-throughput data. Based on the data, people tried to explore molecular characteristics to facilitate predicting the prognosis and improving individualized treatment (Cao et al., 2019; Wang et al., 2019; Niu et al., 2020). Nevertheless, the most appropriate models remain controversial to date. It is widely believed that angiogenesis is highly related to tumorigenesis, metastasis, and migration; anti-angiogenesis therapy has been viewed as a promising treatment for GBM patients (Ahir et al., 2020), but a prediction model concerning angiogenesis has not been developed yet. In the current study, we established an angiogenesis-related gene signature-derived risk score for the first time.

The performance evaluation analysis revealed that the risk score worked well in predicting OS in both the training set and the validation set. The correlations between the risk score and clinical traits were also estimated. Many existing studies demonstrated that non-G-CIMP and IDH wild type were associated with worse prognosis (Tan et al., 2020). Our risk score showed high correlation with G-CIMP and IDH status, which further implied its potential in predicting prognosis.

Tumor microenvironment, as one of the hottest topics, has received extensive attention these past few years. In our study, the high-risk score group revealed a close correlation with type I IFN antiviral response and a higher infiltration proportion of plasmacytoid dendritic cells (pDCs) and neutrophils. It is well-known that both innate and adaptive immune response could promote angiogenesis through releasing pro-angiogenic mediators and then activating endothelial cell proliferation and migration (Ribatti and Crivellato, 2009). The type I IFN family was capable of exerting its anti-tumor activity *via* regulating a wide range of immune cells and inhibiting angiogenesis (Hong et al., 2000; Enomoto et al., 2017; Snell et al., 2017). pDCs, as an important class of antigen-presenting cells (APCs), play a critical role in regulating the immune response to antigens (Megjugorac et al., 2004). Depending on the different microenvironments or stimuli, they could induce either immunogenicity or immune tolerance (Villadangos and Young, 2008; Panda et al., 2017; Waisman et al., 2017). pDC dysfunction induced by impaired IFN-α secretion and upregulation of immune checkpoint mediators was often observed in tumors, including gliomas (Gousias et al., 2013; Aspord et al., 2014; Mitchell et al., 2018). As for neutrophils, they are the most abundant white blood cells in the human circulatory system, involved in the innate immunity (Amulic et al., 2012). Recent studies have demonstrated their complex role in promoting angiogenesis and tumorigenesis (Kim and Bae, 2016; Wu et al., 2020). Both pDCs and neutrophils play a critical role in pro-angiogenesis and immunosuppression (Stockmann et al., 2014; Albini et al., 2018). It is for this reason that our ARG-related risk score has a satisfactory prognostic efficiency.

Chemotherapy, such as anti-angiogenic therapy and immunotherapy, has been expected to be a promising adjunct to traditional surgery and radiotherapy for GBM treatment (Tan et al., 2020). Anti-angiogenic therapy using bevacizumab is a type of targeted anti-cancer therapy that aims to inhibit tumor growth *via* controlling tumor vessel growth. Some studies found that bevacizumab prolonged progression‐free survival in both newly diagnosed and recurrent GBM. On the other hand, not all patients could benefit from it because of its increased toxicity (Chinot et al., 2014; Gilbert et al., 2014). Here, we found that the high-risk group had a higher rate of non-response, indicating that the risk score could serve as an attractive stratification tool to screen suitable patients. Likewise, the risk score was applied to predict the efficiency of immunotherapy. In recent years, there is a growing interest in immunotherapeutic treatments and their intrinsic mechanisms. Though immunotherapy has apparently improved the management of many other tumors, GBM exhibits a high resistance to it (Lim et al., 2018). As discussed above, there is a cross-talk between immune responses and tumor angiogenesis (Albini et al., 2018). Potential response to immune checkpoint blockage therapy was estimated with TIDE algorithm. The results confirmed the potential prognostic efficiency of the risk score on immunotherapy. In general, the risk score we presented would help with providing individualized regimens on GBM.

Furthermore, a nomogram incorporating the angiogenesis-related gene signature, gender, G-CIMP status, IDH status, 1p19q co-deletion status, etc. was generated to predict the OS of GBM. The efficiency was validated by ROC curve. As expected, the performance of the nomogram was satisfactory, which implied a good prospect in clinical practice.

It should be noted that the model has been examined only in a few databases; further validation in multicenter, prospective clinical trials is still needed. Besides, it is derived from 31 genes; the quantity of genes is larger than those of other models, which may hinder its application. Henceforth, we would make further efforts in optimizing and simplifying this angiogenesis-related prognostic model and verifying it in prospective studies.

In the present study, three genes (PLAUR, ITGA5, and FMOD) were finally identified to be key hub genes through construction of a PPI network and screening hub genes with *Cytoscape* software. PLAUR, which is also known as CD87, UPAR, URKR, and U-PAR, is found to be overexpressed in multiple cancers including GBM, and contributes to tumor angiogenesis, cell migration, and invasion (Raghu et al., 2011; Raghu et al., 2012; Schuler et al., 2012; Hu et al., 2015; Loft et al., 2017). It encodes urokinase-type plasminogen activator receptor (uPAR), a GPI-anchored cell membrane receptor, which could bind with urokinase (uPA) and stimulate the intracellular signals associated with tumorigenesis. Raghu et al. validated its over-expression in glioma cell lines and found that specific knockdown of uPA/uPAR could attenuate tumor growing and invasion *via* Notch-1 signaling pathway (Raghu et al., 2011). Besides, some other pro-oncogenic factors like sphingosine-1-phosphate and nitric oxide synthase are also reported to exert their effects through the uPA/uPAR system in glioma (Young et al., 2009; Zhuang et al., 2013). ITGA5, which forms heterodimers together with integrin β1, is known as an important subtype of the integrin *α* chain family. It was validated to be overexpressed in glioma and play a role in predicting prognosis and therapeutic response (Cosset et al., 2012; Blandin et al., 2021; Chen et al., 2021). FMOD was an epigenetically regulated gene, encoding extracellular matrix small leucine-rich proteoglycans. It has been demonstrated to promote cell migration in GBM *via* inducing filamentous actin stress fiber formation, depending on the TGF-β1 pathway (Mondal et al., 2017). It was also suggested to be a mediator in VEGF expression and associated with angiogenesis (Chen et al., 2018). Overall, the current findings indicate that the three genes have a complex relationship with GBM. The underlying mechanisms of the three hub genes demand further explorations.

## Conclusion

In conclusion, we established a reliable angiogenesis-related risk score model that is verified to be effective in predicting the OS and therapeutic responses, suggesting a high likelihood of making individualized treatment strategies for GBM patients. Further studies are needed to optimize the model and explore the inner mechanisms of the key genes in tumorigenesis, metastasis, and migration.

## Data Availability

Publicly available datasets were analyzed in this study. These data can be found here: TCGA-GBM, https://portal.gdc.cancer.gov; CGGA-GBM, http://www.cgga.org.cn; the Repository for Molecular Brain Neoplasia Data (REMBRANDT, http://caintegrator-info.nci.nih.gov/REMBRANDT); and GSE16011 and GSE79671 databases (https://www.ncbi.nlm.nih.gov/geo/).

## Abbreviations

ARG, angiogenesis-related gene; AUC, area under receiver operating characteristic curve; Ang, angiopoietin; bFGF, basic fibroblast growth factor; BP, biological processes; CC, cellular component; CGGA, Chinese glioma genome atlas; DE-ARG, differentially expressed angiogenesis-related gene; GBM, glioblastoma multiforme; G-CIMP, glioma CpG island methylator phenotype; GO gene ontology; HGF, hepatocyte growth factor; IDH, isocitrate dehydrogenases; IFN, interferon; KEGG, Kyoto Encyclopedia of Genes and Genomes; LASSO, least absolute shrinkage and selection operator; MF, molecular function; MMP, matrix metalloproteinase; OS, overall survival; pDC, plasmacytoid dendritic cell; PDGF, platelet-derived growth factor; PPI, protein–protein interaction; ROC, receiver operating characteristic curve; TCGA, The Cancer Genome Atlas; TGF-β, transforming growth factor-β; TIDE, tumor immune dysfunction and exclusion; uPA/uPAR, urokinase/urokinase-type plasminogen activator receptor; VEGF, vascular endothelial growth factor; WHO, World Health Organization.
